# Femtosecond Optical Annealing Induced Polymer Melting and Formation of Solid Droplets

**DOI:** 10.3390/polym11010128

**Published:** 2019-01-13

**Authors:** Jinghui Yang, Cuiying Huang, Xinping Zhang

**Affiliations:** 1 Institute of Information Photonics Technology and College of Applied Sciences, Beijing University of Technology, Beijing 100124, China; b201606021@emails.bjut.edu.cn (J.Y.); huangcy@emails.bjut.edu.cn (C.H.); 2 Basic Course Teaching Department, Chinese People’s Police University, Langfang 065000, China

**Keywords:** femtosecond laser-matter interaction, transient optical annealing, melting polymer droplets, solid-liquid-solid phase transition, molecular rearrangement

## Abstract

Interaction between femtosecond laser pulses with polymeric thin films induced transient optical annealing of the polymer molecules. Melting of the polymer films took place during the transient annealing process, so that a solid-liquid-solid phase transition process was observed. Ultrafast cooling of the melting polymer produced solidified droplets. Microscopic and spectroscopic characterization revealed that the polymer molecules were rearranged with preferable H-aggregation to reach the lowest formation energy during the melting process. Intermolecular coupling was enhanced due to the modified molecular arrangement. This observation of melting of polymeric semiconductors due to the interaction with femtosecond light pulses is potentially important for better understanding laser-matter interactions and for exploring organic optoelectronic devices through special material processing.

## 1. Introduction

Polymeric semiconductors have attracted continuously intense attention in fundamental research and technological applications because of their unique optical and electronic properties and/or advantages [1,2,3]. Efficiencies have been largely improved in polymeric optoelectronic devices with the development of new materials, new designs, and new processing techniques [4,5,6]. Deep insights into the physics, new designs of the device structures, and surface/interfacial processing of the thin-film polymer are always within the important research topics [7,8,9,10,11]. Photophysical and photoelectronic properties of polymer semiconductors can be tailored by changing the fabrication process [7,8,9] or the decorative strategies, such as the annealing processes, the additive solvent treatment, or the laser irradiation [10,11,12]. Poly(9,9-dioctylfluorene-*co*-benzothiadiazole) (F8BT) is a widely investigated and applied light-emitting polymer with excellent efficiency and stability. Light-emitting diodes and optically pumped lasers based on F8BT have been extensively demonstrated [13,14,15,16].

It has been found that polyfluorenes, including F8BT, may be melted into a liquid phase at reasonable heating temperatures. The melting point depends on the molecular weight and longer chains may be melted at higher temperatures, which is between 215 and 300 °C [17,18]. These performances imply the possible tailoring of the F8BT thin films, structures, or devices by such thermal processing. However, such a thermal processing technique is difficult to control and it is very inefficient. Strong interaction between ultrashort laser pulses and polymers have been used to achieve high-quality welding [19,20,21,22,23]. Although a series of nonlinear optical effects may be involved during the interactions [24,25,26,27,28], the laser-pulse-induced thermal process has been the main mechanism for the melting of the polymers. In this paper, we present an optical method to achieve the transient annealing of the F8BT solid film, so that a sequentially solid-liquid-solid phase transition process was observed. Femtosecond laser pulses have been used to accomplish the transient annealing process. Solidified droplets of F8BT were produced after the ultrafast cooling process and quick “freezing” of the liquid phase. Microscopic and spectroscopic characterization of the F8BT droplet structures supplied evidence for the new rearrangement of the molecules with enhanced intermolecular interactions.

## 2. Transient Annealing of Polymeric Thin Films by Femtosecond Laser Pulses

**Figure 1:**
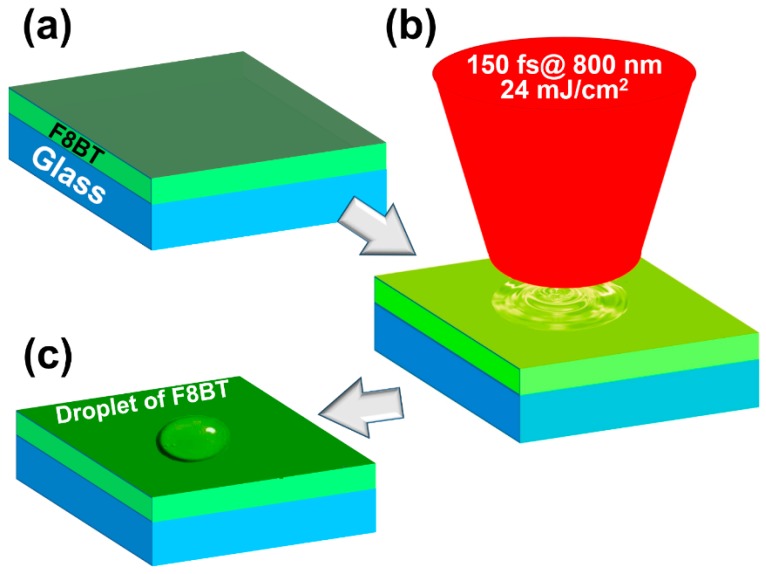
Annealing of the F8BT thin film using femtosecond laser pulses at 800 nm. (**a**) Poly(9,9-dioctylfluorene-*co*-benzothiadiazole) (F8BT) film spin-coated on a glass substrate. (**b**) Laser pulses were focused onto the surface of the F8BT film. (**c**) Production of polymeric droplets.

The thin-film sample of F8BT (American Dye Source, Inc., Quebec, QC, Canada) was prepared by spin-coating the solution of F8BT/chloroform with a concentration of 20 mg/mL onto a glass substrate. The thickness of this pristine film (Figure 1a) was about 90 nm, as measured by an ellipsometer (Ellitop Scientific Co., Ltd., Beijing, China). Femtosecond laser pulses from a Ti:sapphire amplifier (Coherent Inc., Santa Clara, CA, USA) at 800 nm were then sent to the surface of the samples to carry out the transient annealing process, where the laser pulses have a pulse width of 150 fs at FWHM, a repetition rate of 1 kHz, and a maximum pulse energy of 1 mJ. An optical lens with a focal length of 50.8 mm was used to focus laser beam onto the sample, as shown in Figure 1b. The sample was placed about 10 mm before focus, so that the beam size was measured to be about 500 μm in diameter on the surface of the sample. Only a small part of the output of Ti:sapphire amplifier was used for this experiment and the irradiation fluence was around 24 mJ/cm^2^ for each pulse, which was found to be an optimized value for the irradiation fluence after a series of testing experiments. We keep this intensity constant during the transient annealing process, while changing the number of laser pulses for each shot that can reach to samples by the shutter that is placed in the light path. Figure 1c schematically shows the formation of polymeric droplets through transient solid-liquid-solid phase-transition processes during interaction with a specified number of femtosecond pulses. The femtosecond-scale heating-cooling processes are crucial for the transient melting of the polymer film within a local area [19,20,21,22,23]. The number of optical pulses reaching the polymer film can be calculated by *N = T*·*R*, where *T* is the shutter open time in seconds and *R* is the repetition rate of the laser pulse in hertz.

## 3. Results and Discussions

### 3.1. Irradiation-dose Dependence of the Melting Performance

**Figure 2:**
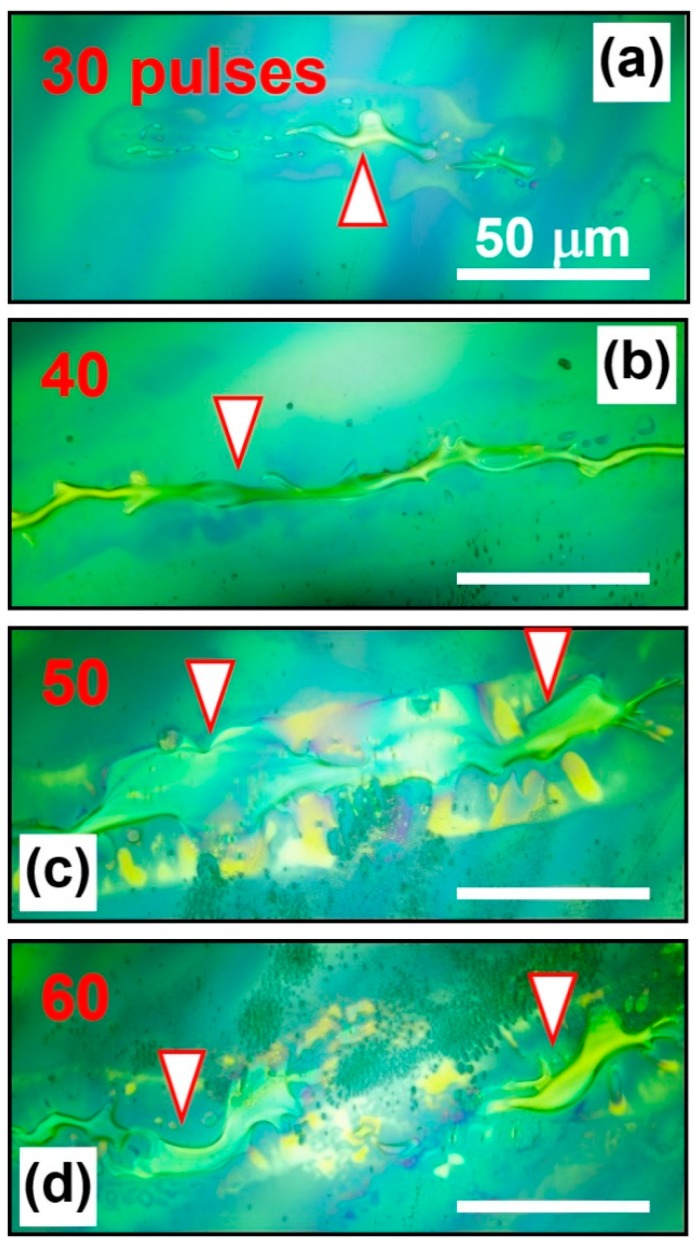
Melted F8BT on the surface of the spin-coated film after being irradiated by femtosecond laser pulses with a number of (**a**) 30, (**b**) 40, (**c**) 50, and (**d**) 60.

Figure 2 shows the optical microscopic images of the annealed thin films of F8BT by different numbers of the femtosecond pulses at 800 nm, where we have used irradiation doses of 30, 40, 50, and 60 pulses. For irradiation by 30 pulses, a small amount of the F8BT film was melted in a small area, as shown in Figure 2a. A small droplet with an irregular shape was produced, as indicated by the triangle in Figure 2a. Apparently, the melted F8BT molecules did not wet the substrate well, so that they aggregate to form a droplet with a large contact angle. However, 30 optical pulses with 1 ms separation corresponds to an open time of roughly 30 ms. Limited by the response speed, the shutter was not able to open to its full diameter, so that only a narrow strip was exposed to the laser beam during irradiation. This mechanisms applies to all of the other results in Figure 2b–d.

When the irradiation dose was increased to 40 pulses, a continuous long strip of the polymer droplet was produced, as shown in Figure 2b and was indicated by the triangle. More solid film was melted within the irradiation area of the laser beam. The melting polymer was frozen quickly after a short interaction. The droplet strip extends in length along the open window of the shutter. For irradiation by 50 pulses, the considerably increased expose time allowed for wider opening of the shutter. Even longer and broader area was irradiated by the laser pulses, so that a much larger area of the polymer film experienced a strong interaction with the laser beam. A much larger droplet strip with much increased width was produced, as shown in Figure 2c and indicated by the two downward triangles. Due to much stronger irradiation, some of the solid film was already burned, which can be identified by the region below the droplet with small particles in dark green.

The damage to the film becomes more serious when the irradiation dose was increased to 60 pulses, as shown in Figure 2d. The major part of droplet was destroyed, so that some small parts remain on the substrate, as indicated by the downward arrows. Therefore, for a pulse fluence of about 24 mJ/cm^2^, the reasonable irradiation dose is from 30 to 60 pulses for the F8BT film on a glass substrate, as verified by Figure 2 and a series of repeated experiments.

We can identify clearly that the structures in Figure 2 are polymeric droplets that are formed through melting and subsequent solidification processes. They are definitely not wrinkling of the polymer film due to mechanical processes during the interaction with the laser pulses. Transient thermal effects are responsible for the formation of these droplets, where similar thermal processes were reported in the welding of polymers using femtosecond laser pulses [19,20,21,22,23]. Furthermore, mechanical wrinkling should have slow-varying edges, however, the droplets have clear edges with smoothly round shapes, as shown in Figure 2 and the scanning electron microscope image in Figure S1. Additionally, as we will discuss in the following sections, the solid droplets are as high as 1 μm, mechanical wrinkling cannot induce such a large modulation on a 90-nm-thick film. Otherwise, the surrounding film would have been torn by such a strong wrinkling, however, we did not observe such effects. Thus, all of the microscopic performance evidenced melting and droplet-forming processes during the interaction between the laser pulses and the polymer film.

The dependence of the melting process on the irradiation dose implies that the solid-to-liquid transition needs a minimum pulse fluence and there is an accumulation effect of the interaction of the laser pulses with the polymer film. However, the accumulation effect does not mean thermal accumulation in the substrate and the polymer film, since the optical pulses are as short as 150 fs and they have a separation of 1 ms. It was reported that solid polymers may be heated up within 9 ns by ultrashort laser pulses and then cooled down to the room temperature within only 14 s [29]. Therefore, there is no chance for thermal accumulation in our experiment.

**Figure 3:**
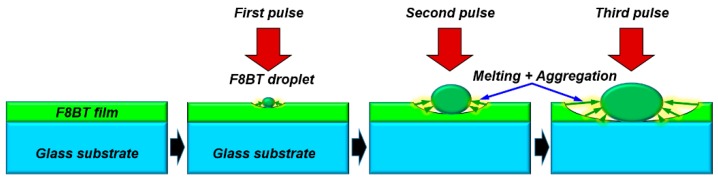
Schematic illustration of the formation mechanisms for the solid droplets of F8BT under different irradiation doses of femtosecond laser pulses.

Thus, the mechanism can be understood as a layer-by-layer melting of the polymer film with successive irradiation by femtosecond pulses. At each pulse irradiation, both surfaces of the previous droplet and the bared film gets melted, and the newly melted layer is aggregated to the droplet to form a larger one. This process is repeated until the whole material within the irradiation area by the laser spot is used up, as depicted schematically in Figure 3. We need to stress that the formation of droplets starts from the first pulse irradiation, as illustrated in Figure 3a, and there is no thermal accumulation during the irradiation by the following pulses. However, the interaction with the following pulses melted more polymers, the aggregation accumulated and enabled the growth of the droplets. Thus, there exists a threshold fluence (μJ/cm^2^) for a single pulse, instead of a threshold number of pulses, although we were able to observe the droplets under the optical microscope after 20-pulse irradiation in our experiment, where the amount or the size of the melted polymers was already large enough. 

When the pulse fluence is lower than the melting threshold of polymer film, the melting process could not occur, no matter how many pulses were used. This is verified by our experimental results in Figure S3, where we have irradiated the F8BT film using 100,000 femtosecond laser pulses with a fluence of 13 mJ/cm^2^. Since the pulse fluence was lower than the melting threshold, we did not observe any melting of the film, although we can identify slight modification on the surface of the film.

If we increase the pulse fluence to 35.2 mJ/cm^2^, melting of the polymer film and the formation of droplets were clearly observed, although we have used only 15 pulses, as shown in Figure S4a. This pulse fluence is apparently above the melting threshold and is even larger the optimized value of 24 mJ/cm^2^. It is also understandable that, even though the droplets were already produced at the first-pulse irradiation, they were too small to be observed under the optical microscope. 

Certainly, over-dose irradiation will burn the molecules from the film and eventually destroy the mechanism for the melting process. As shown in Figure S4b, although we have used only 15 pulses, the F8BT film was burned away at the irradiation by the laser spot with the pulse fluence reached 58.3 mJ/cm^2^. Similar processes and mechanisms have been observed with metals and non-metal materials [30,31,32,33].

### 3.2. Transient Annealing Induced Molecular Re-arrangements in Solid Droplets with Raman Spectroscopic Evidence

**Figure 4:**
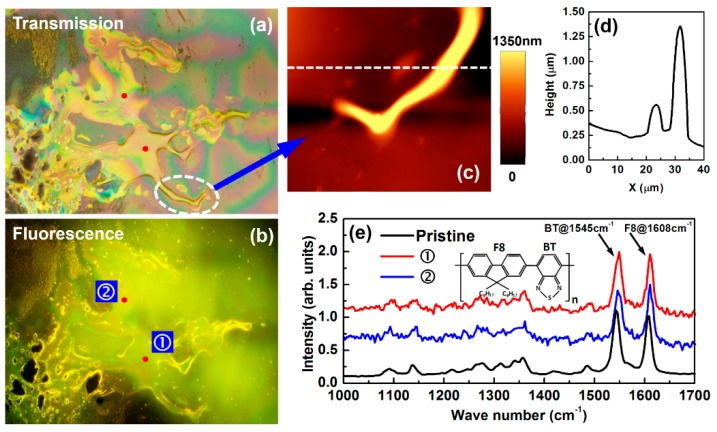
(**a**) and (**b**): Transmission and fluorescence optical microscopic images of a melted area by femtosecond optical pulse annealing. (**c**) Atomic force microscopic (AFM) image of a local area marked in (**a**). (**d**) Plot of the AFM height distribution at a profile indicated by the dashed line in (**c**). (**d**) Comparison between Raman spectra measured on pristine F8BT and annealed droplets of F8BT on sites ① and ② in (**a**) and (**b**). (**e**) Comparison between the Raman spectra measured on the pristine F8BT film, the laser-annealed F8BT droplets on sites ① and ② labeled in (a) and (b).

Figure 4a,b show the transmissive and fluorescence optical microscopic images, respectively, where 30 pulses have been sent to the interaction area for the transient annealing process. The annealed phase or the solidified droplets with irregular shapes can be observed in the optical microscopic images in Figure 4a,b, which were captured by an Olympus fluorescence optical microscope (Olympus Co., Tokyo, Japan) that was equipped with an UV lamp. Figure 4a is the observation of the transmissive image under white-light illumination and Figure 4b is a fluorescence image under excitation by the UV light source. Figure 4c shows the atomic force microscopic (AFM) images that were measured on the samples in Figure 4a, where the dashed ellipse in Figure 4a marks the area for the AFM measurements. Figure 4d shows the height distribution of the cross-sectional profiled of the droplet along the dashed line in Figure 4c. The droplet has a height of about 1.4 μm. Figure 4e shows the Raman spectra measured on two different sites ① (by red curve) and ② (by blue curve), as marked by red spots in Figure 4b. The Raman spectrum measured on pristine F8BT film is also included in Figure 4e by the black curve. A laser beam at 633 nm with a diameter of 1 μm was sent to surface of the sample to excite the Raman emission. Both the AFM microscopic images and the Raman spectra were measured using a WiTec Alpha300S system (WITec Wissenschaftliche Instrumente und Technologie Gmbh, Ulm, Germany).

Two features can be observed dominating the Raman spectrum at about 1545 and 1608 cm^−1^, corresponding to the ring stretching modes of the BT (benzothiadiazole ring stretch) and F8 units (fluorene ring stretch) [34,35]. We cannot identify any big difference between the annealed droplet and the pristine F8BT in the Raman spectrum, implying that the transient annealing did not destroy the molecular structures. Therefore, the transient annealing by femtosecond pulses has mainly led to a phase transition process by melting the sold film and the formation of annealed droplets.

**Figure 5:**
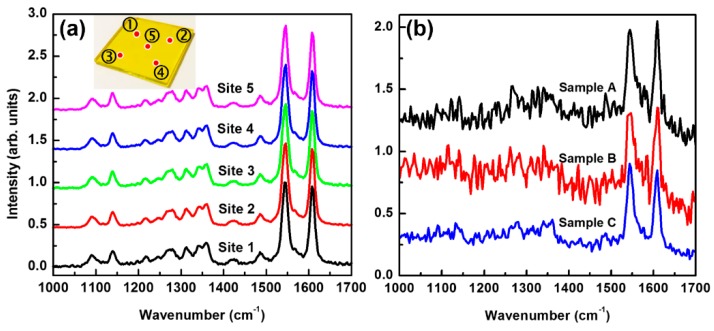
(**a**) Raman spectra measured on five different sites of the pristine F8BT thin film. (**b**) Raman spectra measured on three different droplet samples. Irradiation by 40 pulses has been employed for the production of the droplets used in the measurements.

However, we can still observe variation of the relative intensity between the two features of F8 and BT. For a spin-coated F8BT film, the BT feature was observed always stronger than the F8, as shown in Figure 5a, where Raman spectrum was measured on five different sites. In Figure 5b, we present more measurements on different droplet samples, where we see clearer fluctuation of the two Raman peaks at 1545 and 1608 cm^−1^. In solidified droplets, due to the some flexibility of the “connection” between the F8 and BT units, the rearrangement allows more free orientation of them on the surface of the droplets, leading to the variation of relative intensity of the detected Raman spectral for a fixed excitation and collection direction [36]. This verifies the low-formation rule regulating the formation of the solid droplets.

### 3.3. Photoluminescence Spectroscopic Evidence for the Molecular Rearrangement in the Transient Annealed Polymer Droplets

**Figure 6:**
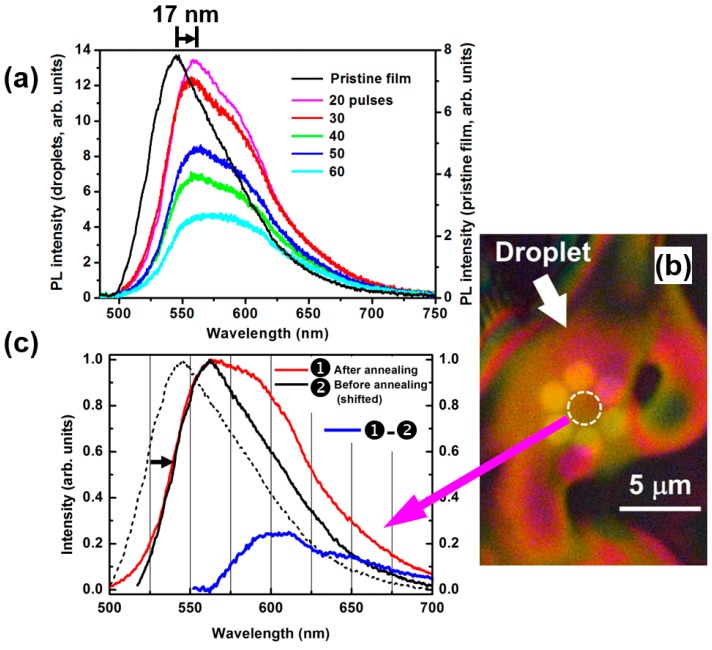
(**a**) PL spectra measured on a pristine F8BT film and on those annealed by 20, 30, 40, 50, and 60 femtosecond pulses. (**b**) Optical microscopic images of the measurement site on the polymer droplet highlighted by a dashed circle, which was produced by irradiation with 40 pulses. (**c**) Red-shifted PL spectrum of pristine F8BT (black, ②), the microscopic PL spectrum of the solidified F8BT droplet (red, ①), and the difference between ① and ② (blue, ①–②). The red spectrum corresponds to an annealing process by 40 pulses. The PL spectrum of pristine F8BT is included as a dashed black curve for comparison.

Figure 6a shows the microscopic photoluminescence (PL) spectra of the F8BT film before (pristine, black) and after (other colors) annealing by different irradiation doses. The method for microscopic PL measurement is illustrated in Figure S2. A Nikon eclipse LV 100 ND optical microscope (Nikon Co., Tokyo, Japan) was modified before it was employed to accomplish the microscopic PL spectroscopic measurements. A laser beam at 470 nm was used as the excitation, which was delivered to the microscope by a single-mode fiber and it was eventually focused to a spot size of about 6 μm in diameter onto the sample. An integration time of 100 ms was used to collect the PL spectrum. Clearly, with increasing the irradiation dose in terms of the number of femtosecond laser pulses from 20 to 40, the PL spectrum becomes broader and slightly red-shifted. The redshift of the spectral peak is as large as 20 nm (from 544 to 564 nm). However, when the number of laser pulses is increased to 50 and 60, the PL spectral bandwidth becomes narrower, suggesting that the sample was over-irradiated. The red-shift and broadening of the melted F8BT have been observed in the direct heating scheme [37,38].

The red-shift and broadening of PL spectra have been explained by the competition between intrachain and interchain electronic coupling processes, which favor J- and H-aggregate behaviors, respectively [39,40,41]. The polymeric π-stacks are inherently two-dimensional (2D) excitonic systems with electronic excitations being delocalized along the polymer chain as well as across neighboring chains, where the π-stack displays a unique set of hybrid photophysical properties [4]. For F8BT, stronger intrachain and weaker interchain interactions lead to larger conjugation lengths and longer exciton motion distance along the polymer chain. During melting of the solid polymer film through ultrafast optical heating, the polymer molecular chains become more flexible and prefer to rearrange to reach lower energy configurations. As a result, the polymer molecules exhibit a J-to-H transition upon solid-liquid-solid phase change and the exciton coherence is spread over two main dimensions: along the polymer backbone and across polymer chains within the stack. These effects enhance the hybridization of the molecular state in both the excited and ground states, so that both the ground- and excited-state bands become broadened. Thus, the bandgap becomes narrowed and the emission spectrum becomes broadened.

In Figure 6c, we show the following spectra: ① the microscopic PL spectrum measured on the annealed F8BT droplet, which was normalized and plotted in red; ② the PL spectrum of the pristine F8BT film, which was normalized, shifted 17 nm to the red, and plotted in solid black; the difference between ① and ② calculated by ①–② plotted in blue. The difference spectrum is peaked at about 600 nm, which approximately characterizes the emission by the interfacial states due to the re-organization of the F8BT molecular chains. The dashed spectrum plots the intrinsic emission from the pristine F8BT as a comparison.

## 4. Conclusions

Under irradiation of femtosecond optical pulses, the thin film of the polymeric semiconductor F8BT experienced a solid-liquid-solid phase transition process. This process was demonstrated and verified by microscopic and spectroscopic performance of the produced solidified “droplets” of F8BT. Rearrangement of the F8BT molecules during the melting process, where they were supplied much more “freedom” for moving in multiple dimensions as compared with the case in solid films, led to looser but more ordered aggregation of the molecules in the “droplets”. Such a rearrangement process of the molecules resulted in the red-shift of the intrinsic PL spectrum and the enhancement of the spectral feature at the long-wavelength tail. This enhancement can be explained by short-range π stacking or H-aggregation with long-range flexibility. Furthermore, this rearrangement is also related to the flexibility between the F8 and BT groups in each F8BT molecule, so that variation in the relative intensity of their respective Raman spectrum has been clearly observed. The discoveries in this work are potentially important for the design and processing of semiconductor materials and devices.

## Supplementary Materials

The supplementary materials are available online at http://www.mdpi.com/2073-4360/11/1/128/s1.
