# Supplementary material for: Femtosecond Optical Annealing Induced Polymer Melting and Formation of Solid Droplets
Source: Polymers (Basel). 2019 Jan 13;11(1):128. doi: 10.3390/polym11010128 (PMC6402037; doi:10.3390/polym11010128)
Supplement: Supplementary file 1 [file polymers-11-00128-s001.pdf]

Femtosecond optical annealing induced polymer melting and formation of solid droplets

Jinghui Yang[1,2], Cuiying Huang[1], and Xinping Zhang[1]*

[1]*Institute of Information Photonics Technology and College of Applied Sciences, Beijing University of Technology, Beijing 100124, P. R. China*

[2]*Basic Course Teaching Department, Chinese People's Police University, Langfang 065000, P. R. China*

**Email: zhangxinping@bjut.edu.cn*

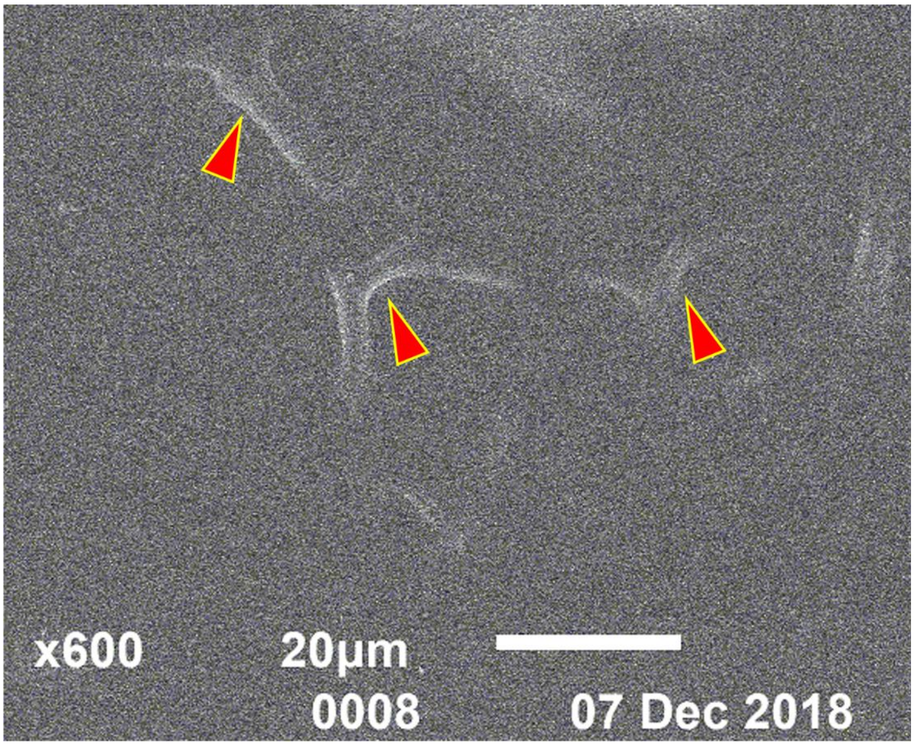

Fig. S1 SEM image of the laser-processed F8BT droplets, as indicated by red triangles.

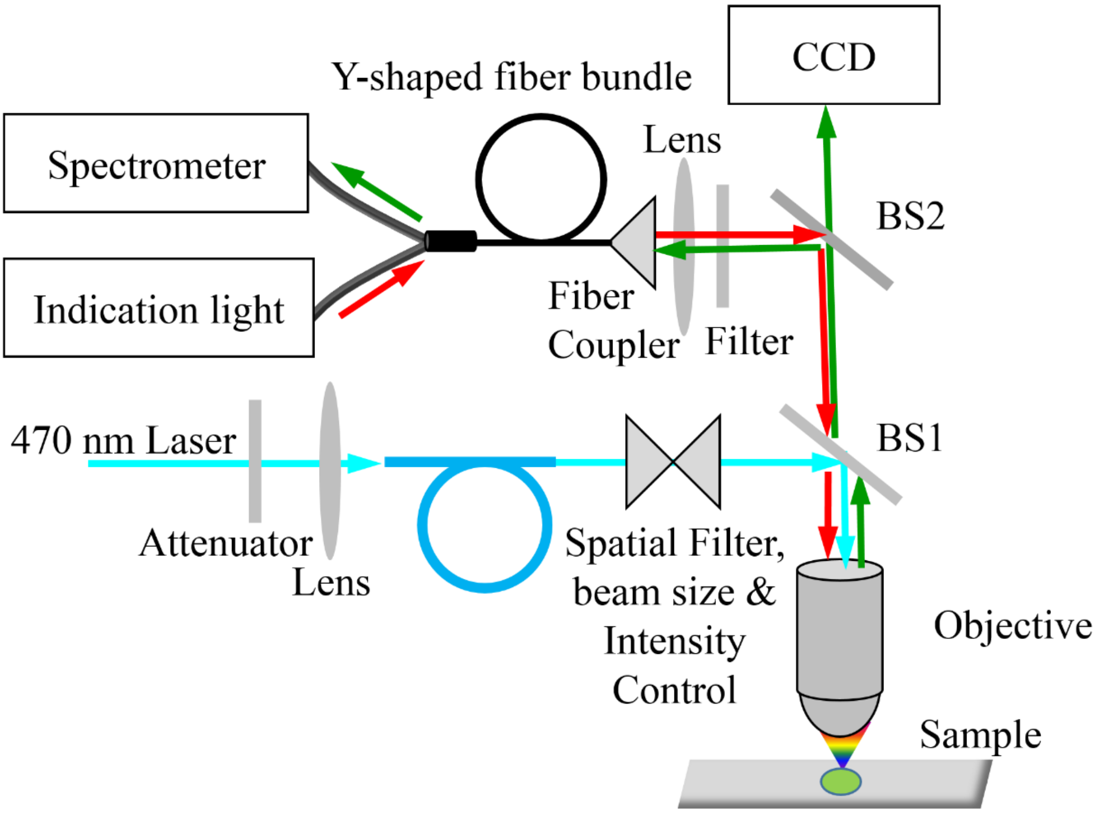

Fig. S2 Illustration of the principles for the microscopic PL measurement. A resolution of 2 μm has been achieved.

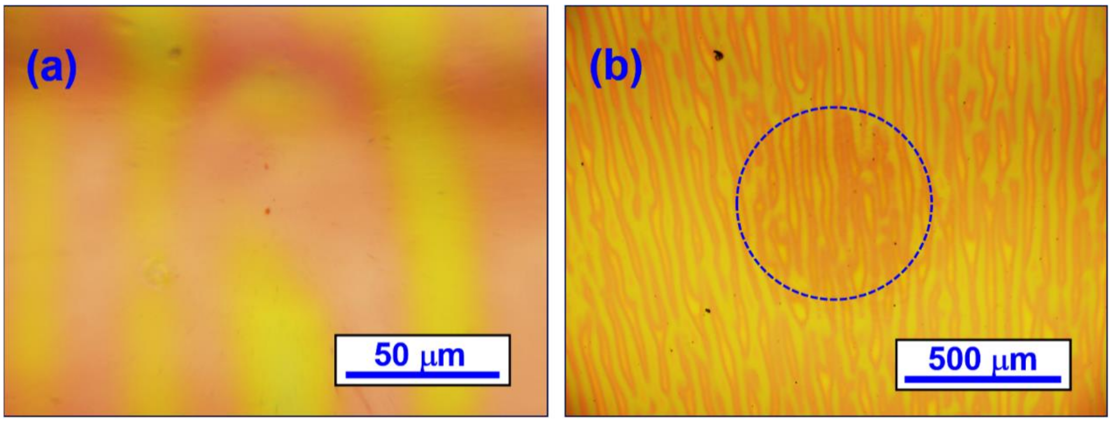

Fig. S3 Modification of surface morphology of spin-coated F8BT film after being irradiated by 10^5 femtosecond laser pulses with a pump fluence of 13 mJ/cm^2: (a) A close view of the modified area and (b) A relatively far field view of the slight modification.

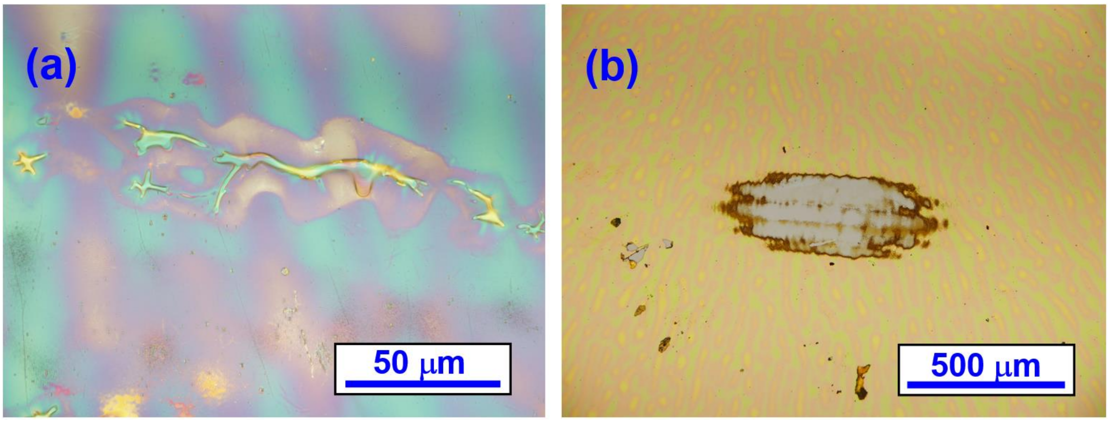

Fig. S4 (a) Molten F8BT droplets on a polymer film after being irradiated by 15 femtosecond laser pulses with a fluence of 35.2 mJ/cm^2. (b) Burned F8BT film after being irradiated by 15 femtosecond laser pulses with a fluence of 58.3 mJ/cm^2.
